# Supplementary material for: Conformal Vortex Crystals
Source: Sci Rep. 2017 Oct 6;7:12766. doi: 10.1038/s41598-017-12807-4 (PMC5630634; doi:10.1038/s41598-017-12807-4)
Supplement: Supplementary file 2 — Supplementary Information [file 41598_2017_12807_MOESM2_ESM.pdf]

Supplementary Information: Conformal Vortex Crystals

Raí M. Menezes[1] and Clécio C. de Souza Silva[1]

[1] *Departamento de Física, Universidade Federal de Pernambuco, Cidade Universitária, 50670-901 Recife-PE, Brazil*

In this Supplementary Information, we present additional simulation results of nonuniform vortex distributions in bulk and thin film superconductors.

SI. CONFORMAL CONFIGURATIONS OF VORTICES IN THIN FILMS

Fig. S1 shows the Voronoi diagram of the typical minimum energy configuration observed in the thin film simulations and its correspondent inverse conformal mapping into the w plane. Apart from some visible deformations, the thin film results present the same arch-pillar configuration as in the bulk case. The topological charge distribution is also similar to that seen in Fig. 1, with high-angle grain boundaries located at the base of each pillar.

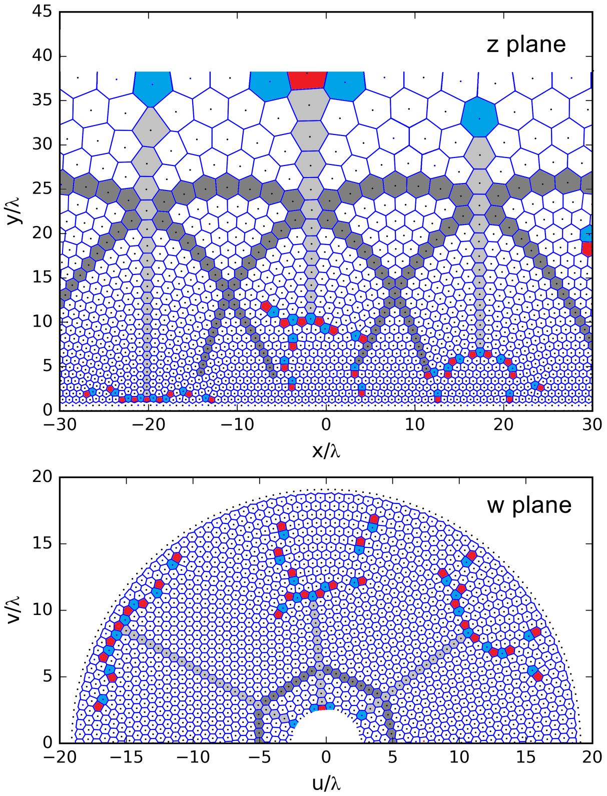

FIG. S1. Top: Typical low-energy configuration of vortices observed in the thin film case under the potential energy shown in Fig. 2-(a) (cyan line). The shaded polygons are defined in the same way as in Fig. 1. Bottom: Inverse conformal map of the physical z plane into the w plane. As in the bulk case, the vertical pillars and arches in the z plane appear in the w plane as, respectively, radial lines and the sides of a regular hexagon. The dots represent the vortex positions, whereas the lines are a Voronoi construction.

After 30 realizations of the annealing process, we found configurations with transverse grain boundaries (TGBs). The distribution of configurations according to the number of TGBs is given in Fig. 2-(c). Note that, in the w plane the deformation in the area of the Voronoi cells near the inner rim is negligible compared with the bulk case. Indeed, the vortex density profile observed for thin films fits better the expected exponential profile in the region $y \geq 15\lambda$ as compared to the bulk case (Fig. 2-(a)), since in the thin film case the distances between the particles are still much smaller than the range of the interaction potential and the continuous approximation is still valid. As in the bulk case, close to the minimum of the external potential, the vortices tend to form a conventional Abrikosov lattice with a principal axis alined with the x axis. This depletion zone is in general larger in the thin film than in the bulk cases. This is consistent with the long range character of the interaction potential, which favors larger correlated domains with the triangular symmetry.

SII. CONFIGURATIONS IN NON-CONFORMAL EXTERNAL POTENTIALS

In Fig. S2 we present results for three different shapes of the external potential, representing the negative concavity (exponential), the linear, and the positive concavity (parabolic) potentials. For the case of vortices in bulk superconductors, all the observed configurations present the basic structure of pillars and arches. Since the exponential potential [Fig. S2-(b)] is conformal for Bessel-interacting vortices, the arch-pillar structure covers the entire system and, apart some topological defects, can be mapped into the w plane as a regular hexagonal lattice (see also Fig. 1). In the linear potential, the vortex distribution [Fig. S2-(e)] presents a more deformed arch structure than in the exponential case. It is similar to the "gravity rainbow" configuration, where the constant force field accommodates a quasi, but not strictly, conformal structure. In Fig. S2-(h), even though the vortex density profile presents a concavity different from the conformal profile, in the higher gradient region the arch-pillar structure can also be observed. Thus, for vortices in bulk samples, the arch-pillar structure was shown as a comfortable and suitable configuration for nonuniform vortex distributions of different shapes. Also note that the observed density profiles for the bulk case fits well the ones expected by the local approximation of Eq. 1 [see Fig. S2-(a, d and g)].

In the thin film case the nonuniform vortex distribution is not observed. As expected, to obtain the nonuniform vortex density profile we need to calculate the specific external

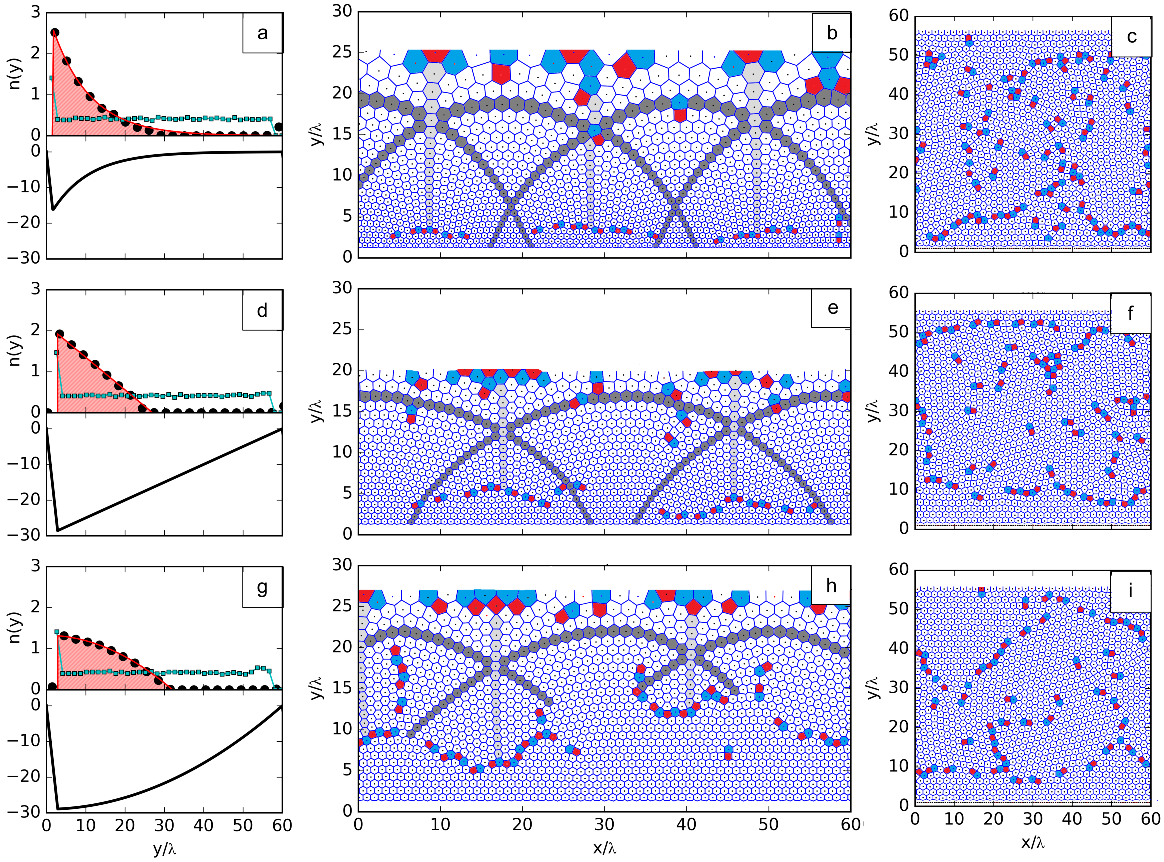

FIG. S2. **Left column** (a, d and g): Top: Observed density profiles for bulk samples (circles) and thin films (squares). The area graph represents the expected density profile for the bulk case for each external potential in accordance with Eq. 1. Bottom: Potential energy profile used in the simulations. **Middle column** (b, e and h) : Voronoi diagram of the typical minimum energy configuration in bulk samples. **Right column** (c, f and i): Voronoi diagram of the typical minimum energy configuration in thin films. The upper (a, b and c), middle (d, e and f), and bottom (g, h and i) panels represent respectively the results obtained for the exponential, linear, and parabolic potentials. The three potentials were chosen in such a way that the vortices were compressed in a region of approximately the same width in y direction $\approx 20\lambda$ against a soft-wall with the same inclination.

potential in accordance with the given vortex-vortex interaction, $V_{int}(\mathbf{r}, \mathbf{r}')$. For the thin film case (Fig. S1) the external potential used is shown in Fig. 2-(a).

SIII. DIFFERENT NUMBER OF VORTICES

We tested whether the conformal vortex crystal can also be found for different vortex densities by changing the number of vortices while keeping the sample size. We present results only for the bulk case. Fig. S4 shows the typical low-energy configuration for $N = 3000$, 2000 and 1000 vortices in a $60\lambda \times 60\lambda$ simulation box after the same annealing procedure described in the main text. The potential energy used is properly adjusted for each number of vortices according to Eq. 2. The CVC structure can be identified in all investigated vortex densities and present the same basic features as the system of Fig. 1.

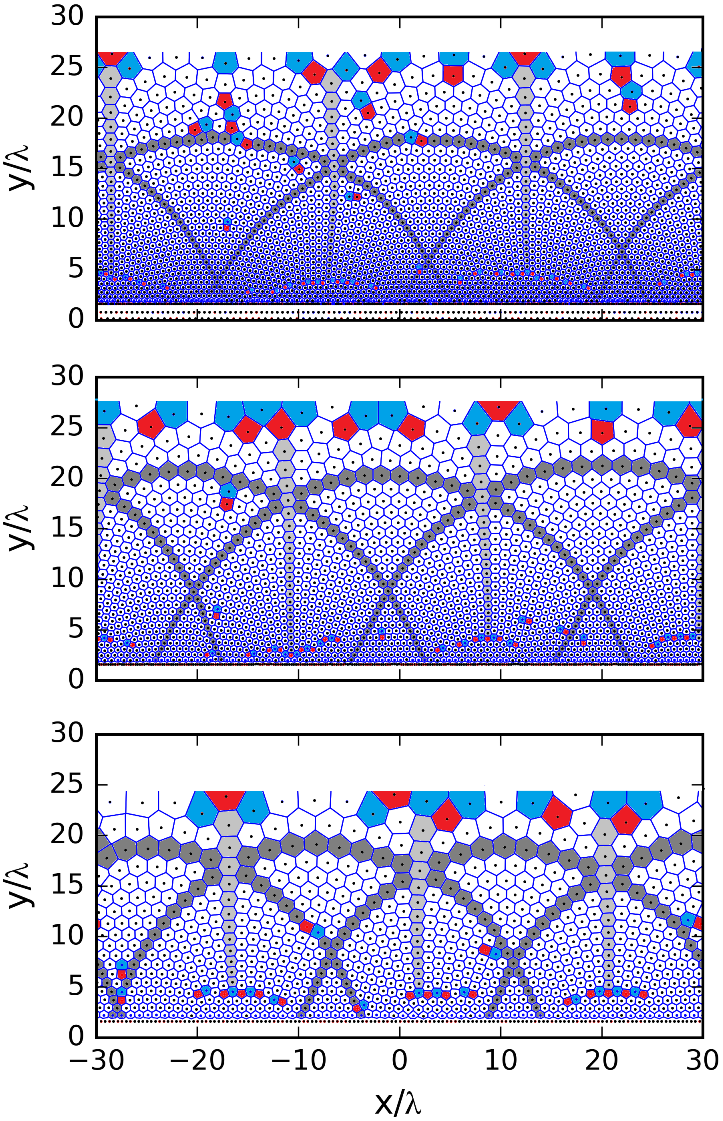

FIG. S3. Typical observed configurations for (a) 3000 vortices. (b) 2000 vortices. (c) 1000 vortices.

SIV. DIFFERENT VALUES OF THE PARAMETER ℓ

A perfect, logarithmic conformal lattice is periodic in the x direction with period $\pi\ell/3$. Therefore, for the conformal crystal to work properly, the commensuration condition $L = p\pi\ell/3$, with p an integer, must be fulfilled. In the main text, we presented results for $p = 3$ ($\ell = L/\pi$), which comprises 3 periods of the conformal structure. In order to check the robustness of the conformal structure and topological charge distribution with respect to other choices of p, we present below typical configurations for a few commensurate (integer p) and one incommensurate value of ℓ ($p = 2.5$). In all cases the conformal structure can clearly be identified with the correct periodicity and the topological charge distribution at the top and bottom of the configurations are essentially the same discussed in the main text for $p = 3$. However, for the incommensurate case, a transverse grain boundary is created in order to fit a non-integer number of periods within the system size L. We changed the number of vortices N concomitantly in order to have the same number of vortices per period of the conformal structure.

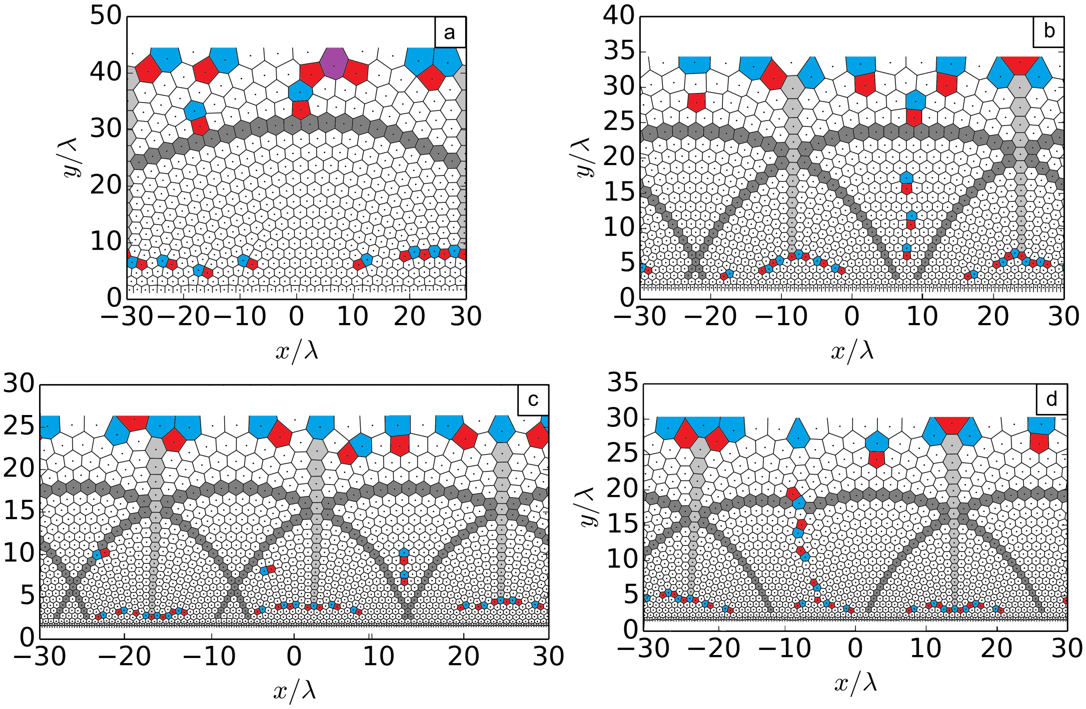

FIG. S4. Typical observed configurations for (a) $N = 500$, $p = 1$, (b) $N = 1000$, $p = 2$, (c) $N = 1500$, $p = 3$, and (d) $N = 1250$, $p = 2.5$.
